# Consequences of Dietary Manganese Deficiency or Mn_2_O_3_ Nanoparticles Supplementation on Rat Manganese Biodistribution and Femur Morphology

**DOI:** 10.3390/nu17193184

**Published:** 2025-10-09

**Authors:** Ewelina Cholewińska, Wojciech Dworzański, Jerzy Juśkiewicz, Piotr Listos, Katarzyna Ognik

**Affiliations:** 1 Department of Biochemistry and Toxicology, Faculty of Animal Sciences and Bioeconomy, University of Life Sciences in Lublin, Akademicka 13, 20-950 Lublin, Poland; katarzyna.ognik@up.lublin.pl; 2 Department of Non-Procedural Clinical Sciences, Faculty of Medical and Health Sciences, Casimir Pulaski Radom University, Bolesława Chrobrego 27, 26-600 Radom, Poland; wojciech686@wp.pl; 3 Clinical Department of Cardiology with Electrotherapy Laboratory, Faculty of Medical and Health Sciences, Casimir Pulaski Radom University, Juliana Aleksandrowicza 5, 26-617 Radom, Poland; 4 Institute of Animal Reproduction and Food Research, Polish Academy of Sciences, Trylińskiego 18, 10-683 Olsztyn, Poland; j.juskiewicz@pan.olsztyn.pl; 5 Department of Pathomorphology and Forensic Medicine, Faculty of Veterinary Medicine, University of Life Sciences in Lublin, Głęboka 30, 20-612 Lublin, Poland; piotr.listos@up.lublin.pl

**Keywords:** manganese(III) oxide, nanoparticles, manganese utilization, bone morphology, femur

## Abstract

**Objectives**: The study aimed to determine the effect of manganese (Mn) exclusion from the dietary mineral mixture and the dietary replacement of the recommended level of MnCO_3_ with Mn_2_O_3_ nanoparticles (Mn_2_O_3_NPs) on the Mn biodistribution and the femur histology. **Methods**: The experiment was conducted on twenty seven Wistar rats divided into three groups (n = 9): a control group receiving the recommended level of Mn (65 mg/kg) in standard form (MnCO_3_); a manganese deficient group (Mn deprived from dietary mineral mixture), and a group receiving diet supplemented Mn_2_O_3_NPs (65 mg/kg) instead of MnCO_3_. During the 12-week experiment, a balance test was performed. After the experiment period, blood and femur were collected from sacrificed rats. The content of Mn in water, diet, urine, feces, plasma, and femur was measured. **Results**: In the Mn-deficient rats, a reduction in Mn intake and excretion, Mn retention index, and blood Mn level, but an increase in Mn digestibility index was noted. In rats supplemented with Mn_2_O_3_NPs, Mn intake and excretion and blood Mn levels were decreased, while Mn retention and digestibility indexes were increased. In both experimental groups, deterioration of femur morphology was noted, but these changes were more severe in the Mn-deficient group. **Conclusions**: The obtained research results indicate that manganese deficiency significantly disturbed the biodistribution of this element and led to the deterioration of the architecture and histological parameters of the femur, emphasizing the key role of manganese in maintaining bone homeostasis. It has also been shown that replacing MnCO_3_ with Mn_2_O_3_NPs allows the maintenance of the correct Mn level in the femur but causes unfavorable changes in its morphology.

## 1. Introduction

Manganese (Mn) is essential for human and animal organisms’ functioning [1,2]. This trace element, as a cofactor of many enzymes, is involved in different metabolic processes, including the neutralization of free radicals (superoxide dismutase MnSOD), ammonia detoxification in the urea cycle (arginase), endogenous synthesis of fatty acids (acetyl-CoA carboxylase), or gluconeogenesis (phosphoenolpyruvate decarboxylase and pyruvate carboxylase) [3]. Manganese is also crucial for the development of the brain, the production of neurotransmitters, and metabolic processes. [2,4]. This microelement is also fundamental to reproductive function, immune responses, hematopoiesis, and blood clotting [2,4,5]. Manganese also enables the proper formation of bone tissue, providing it with the desired structure and strength [6]. It has been proven that Mn participates, among others, in the synthesis of bone matrix and its mineralization, stimulation of osteoblast synthesis and inhibition of the formation of osteoclast-like cells, and regulation of mRNA expression of RANKL receptors or the formation of osteoids [7].

Manganese is relatively poorly absorbed from the diet. The amount of absorbed Mn does not exceed 5% of the total amount contained in the diet [2,8,9,10]. Intestinal absorption of Mn may be additionally hindered by the high degree of diet processing or the presence of other nutrients in it, such as polyphenols, phytates, fiber, or other microelements, with which Mn may interact unfavorably [11]. Consequently, this may increase the risk of Mn deficiency [2]. However, in clinical practice, deficiencies of this element are usually found only in people suffering from various genetic diseases associated with mutations in genes encoding proteins involved in Mn transport and metabolism [12]. In healthy people who consume a diverse, balanced diet, Mn deficiency does not generally occur because this element is present in a wide range of food products, including fruits and vegetables, legumes, cereals, rice, nuts, meat, and seafood, which makes it much easier to meet its demand [13]. Contrastingly, the problem of Mn deficiency is common in laboratory and farm animals, whose diet is much more homogeneous [3]. Although the organism has homeostatic mechanisms that regulate Mn intestinal absorption, distribution to tissues, and excretion [2], which allow it to maintain relatively stable Mn levels in the blood, in the absence or very low Mn dietary intake, especially with long-term administration, these mechanisms may prove insufficient. Consequently, this may lead to deficiency symptoms, including changes in the pattern of macronutrient metabolism, impaired growth, weakening of bone tissue formation processes, reduced fertility, or developmental defects in offspring [2]. Therefore, it is important to supplement Mn in the animals’ diet.

Manganese is most often supplemented as inorganic salts such as MnCO_3_, which can dissociate in the gastrointestinal tract into ionic forms. Mn most often occurs in the intestinal content as divalent cations Mn^2+^ and, slightly less often, as trivalent ones Mn^3+^. It is absorbed most effectively in the initial part of the small intestine [9]. Its penetration into enterocytes occurs by passive diffusion and active transport using membrane transporters, including primarily the divalent metal transporter 1 (DMT1) and the Zip8 and Zip14 transporters [2,8,9,14,15,16]. The absorption of the Mn^3+^ is usually overlapped by complexation with the Tf protein [17]. Mn is then transported from enterocytes to the blood, which probably occurs by mechanisms involving iron export pathways and Mn-binding to transferrin [15]. Mn^2+^ absorbed into the blood can remain free, physically dissolved in the plasma, or, more likely, be bound by citrate and plasma proteins—mainly α2-macroglobulin or albumin. Mn^3+^ is bound by transferrin [16]. Mn^2+^ is then transported to the liver, where it enters via Zip14 transporters located on the basolateral membrane of hepatocytes. Only a part of Mn reaching the liver is further transferred to the general circulation, where it is rebound to carrier proteins such as transferrin, albumin, and α2-macroglobulin [16]. However, this element remains in the plasma relatively short, as it is quickly taken up by tissues—especially the pancreas, kidneys, brain, and bones [5,14,16]. The entry of Mn into the cells of various tissues is a strictly regulated process and occurs with the participation of diverse transporters such as DMT1 or Tf-Tf receptors, choline transporter, citrate transporter, and voltage-gated calcium channels [18]. After entering the cells, Mn is transported to the interior of organelles such as mitochondria (via the calcium uniporter), lysosomes (via ATP13A2), and the Golgi apparatus (via SPCA1) [14]. Mn absorbed by the liver, which did not enter the systemic circulation, is in turn used for the needs of hepatocytes, and its excess is excreted into bile via ZnT-10. Interestingly, however, Zip8 transporters are located in the hepatocyte cell membrane, which enables the recovery of certain amounts of Mn excreted into bile, especially in conditions of Mn deficiency in the organism [16]. Together with bile, Mn then enters the small intestine lumen, where it can be reabsorbed due to enterohepatic recirculation or excreted with feces out of the organism. The transporters involved in the Mn excretion include primarily the ZnT-10 transporter, ferroportin, and NCX (Sodium-Calcium Exchanger) [14]. Available data indicate that fecal excretion is the dominant route of Mn elimination, while renal excretion plays only a secondary role [16,19,20].

Reports have shown that the trace elements’ bioavailability (including Mn) can be modified by the chemical form in which they occur [21,22,23]. Inorganic salts, organic compounds (amino acid chelates), or nanoparticles of the same element often exhibit completely different physicochemical properties that determine their solubility and bioavailability in the gastrointestinal tract [21,22,23]. Kumar et al. [24] report that innovative manganese nanoparticles may have a significant advantage in this aspect over other standard chemical forms of this element. This is primarily because they are characterized by an exceptionally small size and increased biological reactivity, which provides them with increased efficiency in biological systems. Their small size provides them with the ability to penetrate biological membranes and enter cells via alternative routes inaccessible to inorganic salts. The higher biological activity of nanoparticles compared to macro equivalents results largely from the large specific surface area, which is often additionally modified to allow direct and effective contact between nanoparticles and target cells [22]. Increased nanoparticle bioavailability may also result from the fact that in the gastrointestinal tract, they quickly form micelles that are easier to absorb and are excreted in the feces to a lesser extent than their standard macro equivalents [25], which should lead to increased Mn retention, potentially improving their use in the organism.

This study aimed to determine the effect of Mn exclusion from the dietary mineral mixture and the replacement of the recommended level of standard MnCO_3_ (65 mg Mn/kg diet) with innovative Mn_2_O_3_ nanoparticles (Mn_2_O_3_NPs) in the diet on the biodistribution processes of this microelement in the organism and the femur histology. Investigating the impact of these nanoparticles on manganese distribution and bone morphology is essential to evaluate their potential benefits and risks as a novel form of supplementation, given the critical role of manganese in bone development and metabolism.

## 2. Materials and Methods

The experimental design, methodological framework, and experimental procedures presented in this research article have previously been published by Sołek et al. [26] and Różaniecka-Zwolińska et al. [27]. The present experiment is part of a larger project to evaluate the effects of manganese deficiency and supplementation with manganese(III) oxide nanoparticles on the organism’s multi-aspect biological response.

### 2.1. Experimental Animals and Housing

This study involved twenty-seven healthy male outbred Wistar rats (Cmdb:Wi), aged four weeks, obtained from the breeding facility of the Institute of Animal Reproduction and Food Research PAS in Olsztyn, Poland (breeder register 051). Animals were individually housed in stainless steel cages under controlled environmental conditions: the temperature was maintained at 22 ± 1 °C, the relative humidity at 60 ± 10%, a 12 h light/dark cycle was in effect, and ventilation provided 15 air changes per hour. All animal procedures complied with Polish legislation and the European Directive 2010/63/EU on the protection of animals used for scientific purposes [28]. The protocol containing the research questions, experimental schema with in vivo study design and analysis plan was submitted to the Local Ethics Committee for Animal Experiments in Olsztyn for evaluation, and was subsequently approved (No. 13/2022; Olsztyn, Poland, dated 16 March 2022). The animal study followed ARRIVE guidelines [29]. Every effort was made to reduce animal suffering throughout the experiment. During experimental feeding, rats exhibiting adverse signs such as refusal of diet for over two days, prolonged vocalization indicative of pain (exceeding one hour), neurological impairments (e.g., ataxia or inability to maintain posture), or persistent blood in the feces (over 24 h) would be subject to humane euthanasia. A veterinarian employed by the institute determined euthanasia using either gradual carbon dioxide exposure or cervical dislocation following sedation. According to the existing literature and our previous studies, the likelihood of these conditions arising from the dietary nanoparticle treatments was minimal. Consequently, no rats were excluded from the protocol. The project manager was the sole individual aware of the specific allocation of each animal to its respective study group. Additionally, not all analysis contractors were familiar with the treatment animal allocation. Rats serve as an appropriate animal model for manganese research due to several key physiological similarities with humans in manganese absorption, distribution, and metabolism. Rats exhibit similar patterns of manganese biodistribution to target organs, including bones, brain, liver, and kidneys, and demonstrate comparable homeostatic mechanisms for manganese regulation through intestinal absorption and biliary excretion. The rat femur provides an excellent model for studying bone tissue changes, as manganese deficiency in rats produces bone abnormalities characterized by decreased bone density, altered bone metabolism, and impaired osteogenesis that closely parallel human pathophysiology. These translational advantages make rat models particularly valuable for evaluating the safety and efficacy of novel manganese supplementation forms, with findings directly applicable to understanding potential effects in human bone health and mineral metabolism.

### 2.2. Dietary Treatments

The rats were randomly assigned to three groups (n = 9 per group). The number of animals in each group was determined based on our previous research experience. Each rat was treated as an individual experimental unit. Random numbers were generated using the standard =RAND() function in Microsoft Excel. Within the animal housing room, the cages were arranged to ensure that an equal number of rats from each group were positioned in corresponding locations on the cage rack (top, bottom, left side, right side). Over 12 weeks, rats had ad libitum access to tap water and semi-purified diets, formulated according to the American Institute of Nutrition’s recommendations for laboratory rodents (details in Table S1). Diets were stored at 4 °C in hermetic containers. The experimental groups were as follows: control group (K)—received a diet supplemented with 65 mg Mn/kg diet from MnCO_3_ from a mineral mixture; negative control group (B)—received a diet lacking manganese in the mineral mixture; nanoparticle group (N)—received a diet containing 65 mg Mn/kg diet supplied as manganese(III) oxide nanoparticles (Mn_2_O_3_NPs), incorporated as an emulsion with rapeseed oil (standard ingredient used in the preparation of the basic diet) rather than in the mineral mixture for operator safety and achieving better dispersion of nanoparticles throughout the diet. The content of 65 mg Mn/kg diet is based on established rat nutrition guidelines and corresponds to the recommended manganese intake necessary for proper skeletal growth and metabolism in the Wistar model. This level aligns with the AIN-93 standards (American Institute of Nutrition), which specify an optimal range of 30–100 mg Mn/kg diet for rats, thereby avoiding both deficiency and excess of this trace element. By using 65 mg Mn/kg, we ensure a balance of essential enzymatic and mineralization processes in the animals while maintaining comparability of our results with the existing literature. The Mn_2_O_3_NPs were procured from Sky Spring Nanomaterials Inc. (Houston, TX, USA). These nanoparticles were selected for their high purity (99.9%) and uniform physicochemical characteristics, including a manufacturer-specified particle size of 40–60 nm, density of 7.3 g/cm^3^, melting point of 1519 K, and boiling point of 2334 K. Detailed mineral mixture compositions are presented in Tables S2 and S3.

### 2.3. Sample Collection and Monitoring

**Figure 1:**
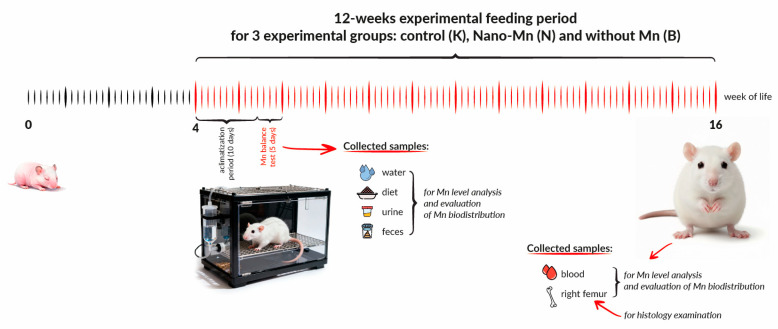
The experimental design scheme includes the timeline and scope of sample collection. Experimental groups: Control (K)—received a diet supplemented with 65 mg Mn/kg diet from MnCO_3_ from a mineral mixture; Nano-Mn (N)—received a diet containing 65 mg Mn/kg diet supplied as manganese(III) oxide nanoparticles (Mn_2_O_3_NPs); Without Mn (B)—received a diet lacking manganese in the mineral mixture.

Following a 10-day acclimation period, feces and urine were collected over five consecutive days from rats (n = 9 per group) housed in individual balance cages (Tecniplast Spa, Buguggiate, Italy) to assess manganese digestibility and utilization (balance test). During the balance test, urine and feces were individually collected from all rats in each experimental group over five consecutive days for further analyses. In addition, the experimental diets and drinking water were also sampled for these analyses. Throughout the study, body weight was recorded at baseline and the end of the experiment, and food intake was monitored daily. During the experiment, no animals from each of the three experimental groups were excluded. At the end of the 12-week experiment, before sacrifice, animals were fasted for 8 h with unrestricted access to water. Anesthesia was induced intraperitoneally with ketamine (100 mg/kg body weight) and xylazine (10 mg/kg body weight) dissolved in 0.9% NaCl. Blood samples were collected from the caudal vena cava into heparinized tubes, followed by euthanasia via cervical dislocation (n = 9 per group). The right femur (n = 9 per group) was excised, segmented, and processed for further analyses: one fragment was decalcified (New Decalc, Medipment, Białystok, Poland) for histological examination, while others were stored at −80 °C. Plasma was separated by centrifugation (350× *g*, 10 min, 4 °C) and frozen at −80 °C. The experimental design scheme, including the schedule and scope of biological samples collected as well as the types of analyses performed, is presented in Figure 1.

### 2.4. Sample Preparation and Manganese Quantification

Samples of diet, drinking water, urine, feces, plasma, and femur tissue underwent microwave-assisted mineralization using a MARSXpress system (CEM, Charlotte, NC, USA). Approximately 0.5 g of each sample was digested with 4 mL of concentrated nitric acid (HNO_3_ Suprapure, Merck KGaA, Darmstadt, Germany). Manganese concentrations were measured by inductively coupled plasma–mass spectrometry (ICP-MS) using a Varian 820-MS instrument (Varian Analytical Instruments, Victoria, Australia), following protocols established by Richardson et al. [30]. Quality control was ensured with certified reference material NIST-1577C Bovine liver (Merck KGaA, Darmstadt, Germany).

### 2.5. Histological Analysis

Femur samples were decalcified in New Decalc solution (Medipment, Białystok, Poland) and fixed in buffered formalin solution at pH 7.4. They were then processed through increasing concentrations of alcohol, acetone, and xylene according to the standard protocol for preparing tissue sections for histopathological evaluation. Bone sections were embedded in paraffin to create a paraffin block using a Leica TP-20 tissue processor (Leica Biosystems, Deer Park, TX, USA), then cut onto a glass slide and subjected to routine hematoxylin and eosin staining (H&E). Morphometric analyses were conducted using a Nikon Eclipse E600 microscope with a Nikon DS-Fi1 digital camera and NIS-Elements BR-2.20 image analysis software (Nikon Europe BV, Amsterdam, The Netherlands).

### 2.6. Statistical Analysis

One-way analysis of variance (ANOVA) followed by appropriate post hoc tests (Tukey’s test) was used to evaluate differences among the three groups—Control (K), Nanoparticle (N), and Manganese-deficient (B). The Tukey test is the standard and recommended post hoc test for all pairwise comparisons when groups are of equal size, as it effectively controls the Type I error rate in multiple testing. Data are presented as means with standard error of the mean (SEM). The statistical analysis was performed using Statistica 13 software (TIBCO Software Inc., Palo Alto, CA, USA).

## 3. Results

### 3.1. Effects of Mn Exclusion from the Mineral Mixture in the Rats’ Diet

**Figure 2:**
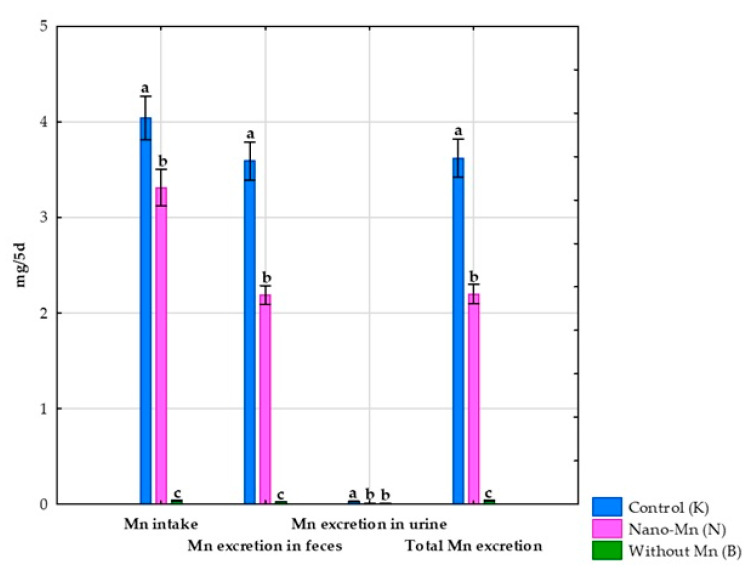
Manganese intake and excretion in experimental rats. ^a–c^ Different superscript letters indicate statistically significant differences between groups (*p* < 0.05).

**Figure 3:**
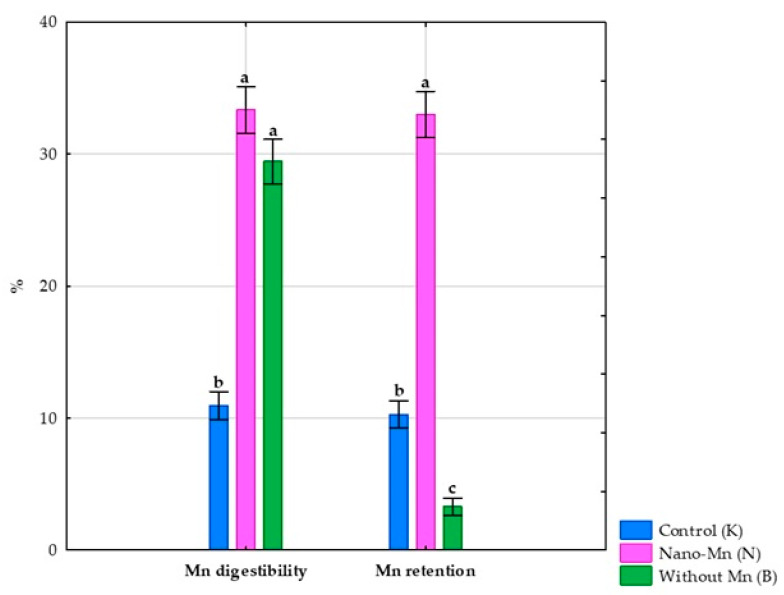
Manganese digestibility and retention in experimental rats. ^a–c^ Different superscript letters indicate statistically significant differences between groups (*p* < 0.05).

**Figure 4:**
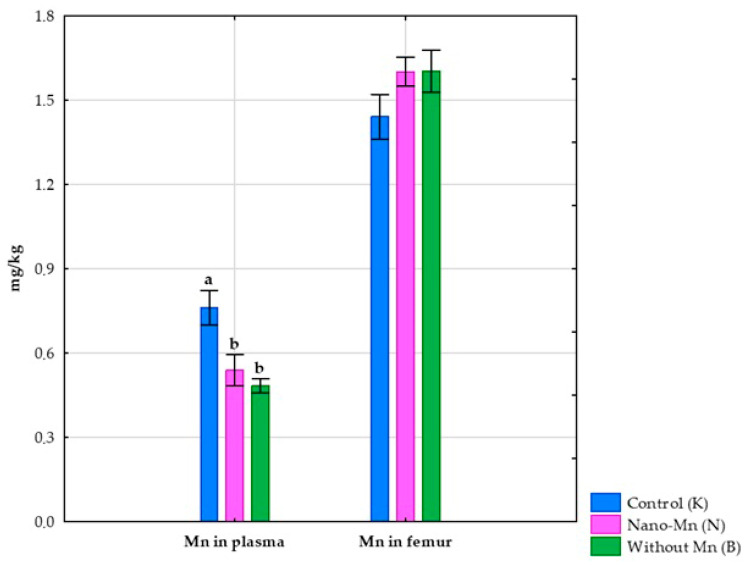
Manganese level in the blood plasma and femur of the experimental rats. ^a–b^ Different superscript letters indicate statistically significant differences between groups (*p* < 0.05).

No effect of using a diet without Mn supplementation in the mineral mixture on the body weight of the tested rats was observed [26]. Complete exclusion of manganese from the mineral mix used in the rats’ diet reduced the intake of this microelement by 99.06% (*p* < 0.001; Table S4) compared to the control group receiving the recommended level of Mn in the standard form of MnCO_3_. This experimental treatment also caused a reduction in Mn excretion both in feces (by 99.25%, *p* < 0.001; Table S4) and in urine (by 96.30%, *p* < 0.001; Table S4), which consequently resulted in a decrease in total Mn excretion from the body (*p* < 0.001; Table S4) by 99.23% compared to the control group (Figure 2). Rats receiving the diet containing the mineral mix without added manganese also showed a 67.97% decrease (*p* < 0.001; Table S4) in the retention index, along with a 168.61% increase (*p* < 0.001; Table S4) in the digestibility coefficient of this microelement compared to rats receiving the recommended level of manganese in the diet in the standard MnCO_3_ form (Figure 3). Compared to the control group, this intervention also decreased the manganese level in the blood plasma of rats (*p* < 0.001; Table S4) by 36.53% (Figure 4).

### 3.2. Effect of Replacing Standard MnCO_3_ with Mn_2_O_3_ Nanoparticles (Mn_2_O_3_NPs) in the Rats’ Diet

Replacing the standard form of Mn (MnCO_3_) with Mn_2_O_3_ nanoparticles in the mineral mixture added to the rat diet did not affect the rats’ body weight [26]. At the same time, this procedure reduced Mn intake by the rats by 18.05% (*p* < 0.001; Table S4) compared to the control group receiving Mn in the standard MnCO_3_ form. Supplementation of the rats’ diet with Mn_2_O_3_ nanoparticles also resulted in decreased Mn excretion both in feces (by 39.09%, *p* < 0.001; Table S4) and urine (by 59.26%, *p* < 0.001; Table S4), which consequently led to a 39.24% reduction in total Mn excretion from the body (*p* < 0.001; Table S4) compared to the control group (Figure 2). Furthermore, rats subjected to this experimental procedure showed a 204.20% higher digestibility index and a 221.01% higher retention index (both *p* < 0.001; Table S4) than the control group receiving a diet containing the mineral mix with the recommended level of Mn in the standard MnCO_3_ form (Figure 3). The addition of the recommended Mn level in the form of Mn_2_O_3_ nanoparticles to the mineral mix instead of MnCO_3_ also caused a 29.04% decrease in Mn levels in the blood plasma of rats (*p* < 0.001; Table S4) compared to the control group (Figure 4).

### 3.3. Effect of Experimental Factors on the Histological Image of the Femur

**Figure 5:**
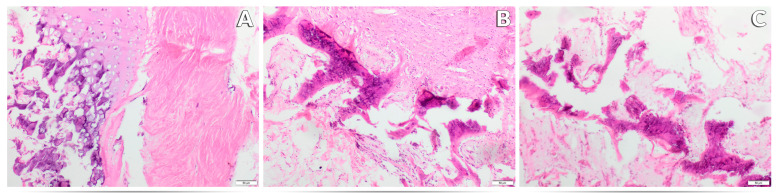
Morphological effects of the recommended level of Mn (65 mg /kg diet) in the form of MnCO_3_—Control (K) (**A**), the recommended level of Mn (65 mg /kg diet) in the form of Mn_2_O_3_ nanoparticles—Nano-Mn (N) (**B**) and diet without Mn in the mineral mixture—Without Mn (B) (**C**) on the femur of rats. ×10 magnification.

**Figure 6:**
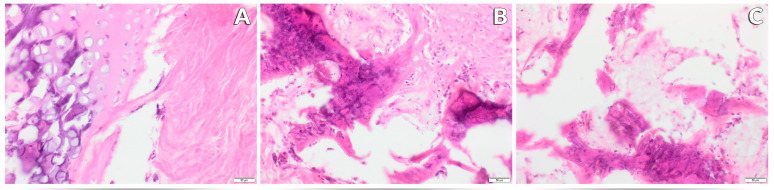
Morphological effects of the recommended level of Mn (65 mg /kg diet) in the form of MnCO_3_—Control (K) (**A**), the recommended level of Mn (65 mg /kg diet) in the form of Mn_2_O_3_ nanoparticles—Nano-Mn (N) (**B**) and diet without Mn in the mineral mixture—Without Mn (B) (**C**) on the femur of rats. ×20 magnification.

In the histopathological evaluation of the bone tissue of rats (femur section) of all three research groups, features of fibrous dystrophy of the compact substance were recognized with varying intensity, characteristic of the individual experimental group. Numerous foci of steatosis were also found inside the marrow cavity and within the compact substance. These changes also had an intensity characteristic for the research group, except for the presence of adipose tissue inside the marrow cavity, which was identical in all groups (Figure 5A–C and Figure 6A–C). The least severe pathological changes were observed in animals receiving the recommended level of Mn in the standard form of MnCO_3_ in the diet (Figure 5A and Figure 6A). Replacing MnCO_3_ with Mn_2_O_3_NPs in the rat diet moderately increased the occurrence of bone fibrosis and increased the features of fat tissue saturation compared to the control group (Figure 5B and Figure 6B). The most severe histological changes in the femur were visible in rats receiving a diet completely devoid of Mn supplementation in the mineral mixture (Figure 5C and Figure 6C).

## 4. Discussion

### 4.1. Manganese Biodistribution and Bone Tissue Alterations in Response to Dietary Manganese Deficiency

The results of the conducted studies showed that the lack of Mn supplementation in the diet significantly reduced the intake of this microelement. In this situation, the body could only use Mn contained exclusively in natural components of the base diet. At the same time, a significant increase in the Mn digestibility coefficient was noted in the studied animals, indicating the intestinal absorption intensification of this microelement. Additionally, in rats fed a Mn-deficient diet, a decrease in the total Mn excretion was observed, with larger Mn amounts being eliminated from the body in the feces than in the urine. This is fully consistent with the available literature data, which indicates that Mn is excreted mainly through the digestive tract via the hepatobiliary pathway [16,19,20]. The results suggest that adaptive compensatory mechanisms have been activated in the organism to increase intestinal absorption efficiency and maximize the retention and use of available Mn to minimize the effects of its deficiency. There are reports that the organism can adapt, at least to some extent, to a deviating supply of Mn in the diet by regulating three key processes for the homeostasis of this element—intestinal absorption, distribution to tissues, and excretion [2]. Therefore, it can be assumed that in conditions of dietary Mn deficiency, the expression of key Mn transporters in intestinal tissue and hepatocytes could have increased, which allowed for a maximum increase in Mn intestinal absorption and its recovery from bile. Interestingly, Choi et al. [31] conducted studies on mice that showed that dietary Mn deficiency increases intestinal permeability by impairing intestinal tight junctions. It cannot be ruled out that intercellular connection conditions of insufficient Mn supply in the diet may be one of the adaptive mechanisms potentially increasing its intestinal absorption by facilitating Mn penetration via an alternative route through intercellular spaces. Interestingly, however, and somewhat contradictory to other research results, the Mn retention index decreased in rats fed a Mn-deficient diet. This indicates that despite the increased absorption of this micronutrient from the digestive tract and the limitation of its elimination from the organism, Mn was not fully retained in the body and used by it because some of it was excreted. Therefore, this may suggest the occurrence of certain disorders or the reorganization of Mn metabolism in a different way than is commonly expected, which prevents permanent management of the absorbed Mn pool. It can be assumed that one of the possible reasons for the observed decrease in Mn retention of the tested rats was certain disorders in the binding and/or transport of Mn to tissues, resulting from limited synthesis or dysfunction of proteins responsible for these processes. Additionally, Mn present in the blood can reach the intestines with it and be excreted via Zip8 and ZnT-10 transporters located in the enterocyte [16], which can probably promote the loss of this element and consequently lead to a retention decrease. Although a reduced Mn level was noted in the blood plasma of the tested rats, which could, to some extent, confirm this assumption, the content of this micronutrient in the femur remained at a normal level. Therefore, the observed reduced plasma Mn level could be because blood is not a Mn reservoir and mainly serves to transport and distribute this element to target tissues, the most important of which seem to be the brain, liver, bones, and pancreas [5]. It is estimated that under conditions of proper dietary Mn supply, its half-life in the blood is only 2 h [32], and in the case of long-term Mn deficiency, as was the case in the rats we studied, the pool of Mn available in the blood could decrease even faster. This assumption seems confirmed by the observed constant and normal level of Mn in the femur of rats receiving a Mn-deficient diet. Additionally, this may suggest that the body prioritizes maintaining the correct level of Mn in the bones, as this micronutrient performs key functions in them, ensuring the proper structure and properties of this tissue. It can be assumed that maintaining Mn homeostasis in bone tissue during Mn deficiency occurs due to increased transcriptomic and proteomic expression of transmembrane proteins—especially Zip14 and Zip8, which are considered to be the most important for Mn ion uptake by bone cells [7]. Nevertheless, Mn deficiency in the diet worsened the processes of bone formation and remodeling, as indicated by the abnormal morphology of this tissue revealed in the histological examination. In the bones of the rats, the presence of fibrous dystrophy of the compact substance and numerous foci of steatosis located both inside the marrow cavity and within the compact substance was found. Although the obtained results are difficult to explain unequivocally, it can be assumed that the main cause of the observed deterioration of bone tissue structure may be functional Mn deficiency. Long-term dietary Mn deficiency may likely result in its “blocking” in bone forms or structural regions that are not directly accessible to osteoblasts and osteoclasts. As a result, this may significantly limit the availability of “functional” Mn for bone repair and metabolic processes. Impairment of proper osteoblast activity due to functional Mn deficiency may probably increase osteoclast-dependent bone resorption processes, thus disturbing the balance between synthesis and lysis of bone tissue, which, with additionally impaired mineralization, may prevent the deposition of properly mineralized bone matrix. As a consequence, fibrous dystrophy of the compact substance may develop, meaning the deposition of fibrous connective tissue as a form of provisional repair of emerging bone defects caused by excessive bone resorption, about its synthesis. The suggested functional Mn deficiency also seems to lead to a change in the profile of bone marrow matrix cells towards adipogenesis. There are reports that, as a result of the stress factor (and Mn deficiency is undoubtedly one such factor), bone marrow mesenchymal stem/stromal cells (BMSC) receive signals that initiate changes in their transcriptional profile, cellular metabolism, and morphology toward their differentiation into adipocytes (fat cells) instead of normal osteoblasts [33]. As a result, steatosis of the marrow cavities and compact substance may occur, as was the case in the femur of the rats we studied receiving a Mn-deficient diet.

### 4.2. Manganese Biodistribution and Bone Tissue Alterations in Response to Dietary Mn_2_O_3_ Nanoparticles Supplementation

The results of the conducted studies showed that replacing the recommended level of MnCO_3_ with Mn_2_O_3_NPs in the mineral mixture added to the rat diet significantly reduced the amount of Mn excreted in feces and urine, which in turn translated into an increase in both the retention and digestibility index. This indicates that the body retains and uses Mn supplied in the form of nanoparticles more effectively than standard inorganic salt. This suggests that nanoparticles can be absorbed from the gastrointestinal tract more effectively than their ionic counterparts because, in addition to standard transport using standard membrane transporters, they can probably also be absorbed via alternative routes, including endocytosis or transport through paracellular spaces [34]. Research conducted by Singh et al. [35], who administered 30, 300, or 3000 mg/kg per day of MnO_2_-NPs (<30 nm) orally to Wistar rats for 28 days, showed a dose-dependent increase in the Mn level excreted from the body in urine and feces, which indicates that the improvement in the retention and utilization of nanoparticle Mn can only be achieved within a specific dose range. In the case of increased exposure to this microelement, the body intensifies the processes of its elimination, which is probably the result of the activation of adaptive mechanisms that allow for the maintenance of Mn homeostasis and protect the organism against its excessive accumulation. Singh et al. [35] also noted a significant increase in the Mn level in both blood plasma and tissues such as the liver, brain, heart, kidneys, and spleen in rats receiving MnO_2_-NPs, and this effect was additionally enhanced by the increase in the used dose. The results of our studies showed that replacing MnCO_3_ with Mn_2_O_3_NPs in the rat diet reduced the Mn level in the plasma, while it had no significant effect on the content of this element in the femur. The differences between the results noted in our studies and Singh et al. [35] may probably result primarily from the level of nanoparticles used, and it is also possible that it may be due to the chemical form used (Mn_2_O_3_NPs vs. MnO_2_-NPs). However, the obtained results allow us to assume that Mn_2_O_3_NPs may show an increased affinity for target tissues than traditional ionic forms, which consequently contributed to their effective distribution in the body and thus translated into a reduction in the Mn level in the blood. Although the mechanism of translocation and Mn nanoparticle uptake by tissue cells is not yet well understood, one theory assumes that this may occur due to the dissociation of insoluble and charge-neutral nanoparticles into easily migrating Mn ions [36]. It has been shown that Mn nanoparticles present in the blood can penetrate macrophage phagosomes by endocytosis, and the acidic pH promotes the release of Mn ions from their surface. As a result, neutral nanoparticles become Mn ion carriers, allowing them to penetrate the cell, which is not possible in the case of a particle originally endowed with an electrical charge [36]. Oszlánczi et al. [37] based on the results of studies in which rats received MnO_2_ nanoparticles found that the mentioned mechanism allows the Mn nanoparticles to cross the blood–brain barrier and penetrate the cells of nervous tissue, hence it is possible that it also plays an important role in the penetration of nanoparticles into other tissues. Although bone is one of the tissues that captures the largest amounts of available Mn, the presented studies did not show Mn excessive accumulation under the conditions of dietary replacement of the recommended level of MnCO_3_ with Mn_2_O_3_NPs. Nevertheless, despite the correct physiological Mn content found in the femur, the inclusion of Mn_2_O_3_NPs in the rat diet harmed the morphology of the tissue, causing moderate fibrosis and saturation with adipose tissue. This is somewhat surprising because the available literature contains many reports of the positive effect of Mn nanoparticles on bones. Yang et al. [38] demonstrate that supplementing resistant bone defects with a scaffold consisting of poly-L-lactide (PLLA) and MnO_2_ nanoparticles (MnO_2_NPs) supports regeneration processes, as MnO_2_NPs, exhibiting strong antioxidant properties, effectively remove endogenous reactive oxygen species. Moreover, MnO_2_NPs significantly alleviate the inflammatory response by promoting the M2 macrophages polarization and reprogramming the osteoimmune microenvironment conducive to angiogenesis and osteogenesis. Additionally, they also promote osteogenic differentiation as a result of the upregulation of the TGF-β/Smad signaling pathway in bone marrow stem cells (hBMSCs). Kumar et al. [39] indicate that MnO_2_ nanoparticles may also have a beneficial effect on cartilage tissue, effectively protecting it from oxidative stress. Therefore, it is somewhat difficult to explain the toxicity of Mn_2_O_3_NPs towards femoral tissue observed in our study. However, it can be assumed that the observed deterioration of bone tissue structure may result from the ability of Mn_2_O_3_NPs to induce oxidative stress. Although the antioxidant function of manganese, among others, as a cofactor of superoxide dismutase (Mn-SOD), is widely known and well documented, this element, belonging to the group of transition metals, under certain conditions may also act as a pro-oxidant, generating the formation of cytotoxic levels of free radicals during the redox cycle [40]. In the case of specific Mn nanoparticles or their compounds, this effect can be even more intense, which has been confirmed in numerous in vivo and in vitro studies [37,41,42,43,44,45,46,47,48]. Therefore, it can be assumed that the Mn_2_O_3_NPs used in our experiment contributed to the occurrence of oxidative stress in bone cells, which consequently led to the disruption of metabolic processes and inflammation, resulting in fibrosis-like changes and deterioration of bone structure and properties. Chen et al. [14] indicate that Mn and its compound nanoparticles can also disrupt protein folding and induce endoplasmic reticulum (ER) stress, autophagy dysregulation, and apoptosis. Presumably, Mn_2_O_3_NPs introduced into the body can disrupt functions and structure also by modulating key signaling pathways regulating bone cell activity (Wnt/β-catenin, RANK/RANKL/OPG or MAPK) and directly interacting with genetic material and extracellular components, which consequently can lead to gene expression and epigenetic changes and abnormal mineralization, respectively. There are also reports that Mn, especially at higher concentrations, can compete with other elements (especially divalent ones) for membrane transport channels [49], thereby limiting their bioavailability. Therefore, it is probable that the Mn_2_O_3_NPs used in the rat diet, which have a greater affinity for non-specific transport and binding proteins than other microelements, especially Ca and P, which are particularly important for the functioning of bone tissue, could have significantly hindered their penetration into cells, which consequently led to disturbances in bone metabolic processes and prevented proper mineralization of the bone matrix.

Despite their utility, rat models have inherent limitations when translating bone histology findings to humans. Rats exhibit a markedly faster bone remodeling cycle than humans, which can exaggerate the severity of femoral changes observed under Mn deficiency or nanoparticle exposure. Additionally, interspecies differences in gastrointestinal physiology and microbiota composition can alter Mn absorption and bioavailability, potentially affecting bone morphology outcomes. Finally, healthy laboratory rats lack the diverse comorbidities present in human populations, which may limit the model’s ability to predict clinical responses to Mn supplementation or deficiency in patients with underlying diseases.

## 5. Conclusions

To conclude, the obtained research results indicate that manganese deficiency significantly disturbed the biodistribution of this element and led to the deterioration of the architecture and histological parameters of the femur, emphasizing the key role of manganese in maintaining bone homeostasis. The obtained research results have also shown that Mn_2_O_3_NPs are characterized by higher bioavailability and are retained in the body to a greater extent than standard MnCO_3_. However, replacing the recommended level of MnCO_3_ with Mn_2_O_3_NPs allows the maintenance of the correct level of this microelement in the femur but also causes unfavorable changes in its morphology. The results suggest that introducing Mn_2_O_3_NPs to the diet as a potential supplement is unfavorable because it carries the risk of bone tissue damage.

## Data Availability

The original contributions presented in this study are included in the article/Supplementary Materials. Further inquiries can be directed to the corresponding authors.

## Supplementary Materials

The following supporting information can be downloaded at: https://www.mdpi.com/article/10.3390/nu17193184/s1, Table S1: Composition of basal experimental diet fed to rats; Table S2: Experimental schema; Table S3: Composition of mineral mixtures (MX) used in experimental diets; Table S4: Manganese balance test. References [50,51] are cited in the Supplementary Materials.

## Abbreviation

The following abbreviation is used in this manuscript:

Mn_2_O_3_NPs	Mn_2_O_3_ nanoparticles
