# Supplementary material for: Consequences of Dietary Manganese Deficiency or Mn2O3 Nanoparticles Supplementation on Rat Manganese Biodistribution and Femur Morphology
Source: Nutrients. 2025 Oct 9;17(19):3184. doi: 10.3390/nu17193184 (PMC12526447; doi:10.3390/nu17193184)
Supplement: Supplementary file 1 [file nutrients-17-03184-s001.zip › Supplementary material - Table S1.pdf]

Table S1. Composition of basal experimental diet fed to rats, % (This table is also included in the articles published by Sołek et al. [1] and Różaniecka-Zwolińska et al. [2].)

Ingredient	Content
**Unchangeable ingredients**	
Casein[1]	14.8
DL-methionine	0.2
Cellulose[2]	8.0
Choline chloride	0.2
Rapeseed oil	8.0
Cholesterol	0.3
Vitamin mix[3]	1.0
Maize starch[4]	64.0
**Changeable ingredient**	
Mineral mix (MX)[5]	3.5
**Calculated content**	
Crude protein	13.5

Notes: [1]Casein preparation: crude protein 89.7%, crude fat 0.3%, ash 2.0%, and water 8.0%. [2]α-Cellulose (SIGMA, Poznan, Poland), the main source of dietary fibre. [3]AIN-93G-VM [3], g/kg mix: 3.0 nicotinic acid, 1.6 Ca pantothenate, 0.7 pyridoxine-HCl, 0.6 thiamin-HCl, 0.6 riboflavin, 0.2 folic acid, 0.02 biotin, 2.5 vitamin B-12 (cyanocobalamin, 0.1% in mannitol), 15.0 vitamin E (all-rac-α-tocopheryl acetate, 500 IU/g), 0.8 vitamin A (all-trans-retinyl palmitate, 500000 IU/g), 0.25 vitamin D-3 (cholecalciferol, 400000 IU/g), 0.075 vitamin K-1 (phylloquinone), 974.655 powdered sucrose. [4]Maize starch preparation: crude protein 0.6%, crude fat 0.9%, ash 0.2%, total dietary fibre 0%, and water 8.8%. [5]Changeable dietary ingredient to manganese level; mineral mixture (the base according to NRC [4]) with standard Mn level and deprived of Mn, see Tables S2 and S3.

[1] Sołek, P.; Różaniecka, K.; Juśkiewicz, J.; Fotschki, B.; Stępniowska, A.; Ognik, K. Consequences of dietary manganese-based nanoparticles supplementation or deficiency on systemic health and gut metabolic dynamics in rats. *Nanotechnol. Sci. Appl.* **2025**, 18, 19–34. https://doi.org/10.2147/NSA.S494533

[2] Różaniecka-Zwolińska, K.; Cholewińska, E.; Fotschki, B.; Juśkiewicz, J.; Ognik, K. Manganese deficiency or dietary manganese(III) oxide nanoparticle supplementation: consequences for hematology, and intestinal and brain immunity in rats. *Front. Immunol.* **2025**, 16, 1528770. https://doi.org/10.3389/fimmu.2025.1528770

[3] Reeves, P.G. Components of the AIN-93 diets as improvements in the AIN-76A diet. *J. Nutr.* **1997**, 127(5 Suppl), 838S–841S. https://doi.org/10.1093/jn/127.5.838S

[4] National Research Council. Science and the Endangered Species Act. Washington, DC: National Academies Press; 1995.
