# Supplementary material for: Consequences of Dietary Manganese Deficiency or Mn2O3 Nanoparticles Supplementation on Rat Manganese Biodistribution and Femur Morphology
Source: Nutrients. 2025 Oct 9;17(19):3184. doi: 10.3390/nu17193184 (PMC12526447; doi:10.3390/nu17193184)
Supplement: Supplementary file 1 [file nutrients-17-03184-s001.zip › Supplementary material - Table S2.pdf]

Table S2. Experimental schema*(provided manganese dosage was calculated taking into account $MnCO_3$ in MX or Mn from Mn_2O_3 nanoparticles preparation) (This table is also included in the articles published by Sołek et al. [1] and Różaniecka-Zwolińska et al. [2].)

Group	12 weeks of feeding
**B** (Negative CONT, without Mn in MX)	A diet with MX deprived of Mn (n=9)
**K** (Control, with standard supplementation of Mn in MX)	A diet containing 65 mg/kg Mn from $MnCO_3$ (n=9)
**N** (Nano Mn, with standard supplementation of Mn but from novel nanoparticle source)	A diet containing 65 mg/kg Mn from Mn_2O_3 nanoparticles (n=9)

Notes: n=9, number of rats used in a particular feeding period. *Experimental groups: B – during all twelve weeks of feeding the Mn deficient rats were given a diet with MX deprived of Mn ($MnCO_3$ excluded from MX); K – the rats were fed a diet with standard mineral mixture (MX) resulting in 65 mg Mn (from $MnCO_3$ in MX) per 1 kg of a diet during 12 weeks of feeding; N – the rats were given a diet containing 65 mg/kg Mn from Mn_2O_3 nanoparticles preparation per 1 kg of a diet during 12 weeks of feeding.

[1] Sołek, P.; Różaniecka, K.; Juśkiewicz, J.; Fotschki, B.; Stępniowska, A.; Ognik, K. Consequences of dietary manganese-based nanoparticles supplementation or deficiency on systemic health and gut metabolic dynamics in rats. *Nanotechnol. Sci. Appl.* **2025**, 18, 19–34. https://doi.org/10.2147/NSA.S494533

[2] Różaniecka-Zwolińska, K.; Cholewińska, E.; Fotschki, B.; Juśkiewicz, J.; Ognik, K. Manganese deficiency or dietary manganese(III) oxide nanoparticle supplementation: consequences for hematology, and intestinal and brain immunity in rats. *Front. Immunol.* **2025**, 16, 1528770. https://doi.org/10.3389/fimmu.2025.1528770
