# Supplementary material for: Consequences of Dietary Manganese Deficiency or Mn2O3 Nanoparticles Supplementation on Rat Manganese Biodistribution and Femur Morphology
Source: Nutrients. 2025 Oct 9;17(19):3184. doi: 10.3390/nu17193184 (PMC12526447; doi:10.3390/nu17193184)
Supplement: Supplementary file 1 [file nutrients-17-03184-s001.zip › Supplementary material - Table S3.pdf]

**Table S3.** Composition of mineral mixtures (MX) used in experimental diets (This table is also included in the articles published by Sołek et al. [1] and Różaniecka-Zwolińska et al. [2].)

| | **MX with standard Mn dosage[1]** | **MX deprived of Mn[2]** |
|---|---|---|
| Calcium carbonate anhydrous $CaCO_3$ | 357 | 357 |
| Potassium phosphate monobasic $K_2HPO_4$ | 196 | 196 |
| Potassium citrate $C_6H_5K_3O_7$ | 70.78 | 70.78 |
| Sodium chloride NaCl | 74 | 74 |
| Potassium sulphate $K_2SO_4$ | 46.6 | 46.6 |
| Magnesium oxide MgO | 24 | 24 |
| **Microelements mixture** | 18 | 18 |
| Starch | To 1000g = 213.62 | To 1000g = 213.62 |
| **Microelements mixture:** | | |
| Ferric citrate [16,7% Fe] | 31 | 31 |
| Zinc carbonate $ZnCO_3$ [56% Zn] | 4.5 | 4.5 |
| Manganous carbonate $MnCO_3$ [44.4% Mn] | 23.4 | 0 |
| Copper carbonate $CuCO_3$ [55.5% Cu] | 1.85 | 1.85 |
| Potassium iodate KJ | 0.04 | 0.04 |
| Citric acid $C_6H_8O_7$ | To 100 g = 39.21 g | To 100 g = 62.61 |

**Notes:** [1] given to the K group (12 weeks of feeding), [2] given to the B and N groups (12 weeks of feeding), but the N group was provided with the appropriate amount of Mn from $Mn_2O_3$ nanoparticles preparation as an emulsion along with dietary rapeseed oil

[1] Sołek, P.; Różaniecka, K.; Juśkiewicz, J.; Fotschki, B.; Stępniowska, A.; Ognik, K. Consequences of dietary manganese-based nanoparticles supplementation or deficiency on systemic health and gut metabolic dynamics in rats. *Nanotechnol. Sci. Appl.* **2025**, 18, 19–34. https://doi.org/10.2147/NSA.S494533

[2] Różaniecka-Zwolińska, K.; Cholewińska, E.; Fotschki, B.; Juśkiewicz, J.; Ognik, K. Manganese deficiency or dietary manganese(III) oxide nanoparticle supplementation: consequences for hematology, and intestinal and brain immunity in rats. *Front. Immunol.* **2025**, 16, 1528770. https://doi.org/10.3389/fimmu.2025.1528770
