# Supplementary material for: Consequences of Dietary Manganese Deficiency or Mn2O3 Nanoparticles Supplementation on Rat Manganese Biodistribution and Femur Morphology
Source: Nutrients. 2025 Oct 9;17(19):3184. doi: 10.3390/nu17193184 (PMC12526447; doi:10.3390/nu17193184)
Supplement: Supplementary file 1 [file nutrients-17-03184-s001.zip › Supplementary material - Table S4.pdf]

Table S4. Manganese balance test.

	Experimental groups			SEM	*P*-value
	K (Control)	N (Nano-Mn)	B (Without Mn)		
Mn in plasma, mg/kg	0.761[a]	0.540[b]	0.483[b]	0.036	<0.001
Mn in femur, mg/kg	1.441	1.601	1.603	0.041	0.116
**Balance 5-day test:**					
Mn intake, mg/5d	4.039[a]	3.310[b]	0.038[c]	0.354	<0.001
Mn excretion in feces, mg/5d	3.592[a]	2.188[b]	0.027[c]	0.296	<0.001
Mn excretion in urine, mg/5d	0.027[a]	0.011[b]	0.001[b]	0.002	<0.001
Total Mn excretion in feces and urine, mg/5d	3.619[a]	2.199[b]	0.028[c]	0.298	<0.001
Mn digestibility, %	10.96[b]	33.34[a]	29.44[a]	2.096	<0.001
Mn retention, %	10.28[b]	33.00[a]	3.293[c]	2.580	<0.001

Notes: SEM, pooled standard error of mean (standard deviation for all rats divided by the square root of rat number, n=27); [a-c] Mean values within a row with unlike superscript letters are shown to be significantly different ($P<0.05$). Groups: B – during all twelve weeks of feeding the Mn deficient rats were given a diet with MX deprived of Mn ($MnCO_3$ excluded from MX); K – the rats were fed a diet with standard mineral mixture (MX) resulting in 65 mg Mn (from $MnCO_3$ in MX) per 1 kg of a diet during 12 weeks of feeding; N – the rats were given a diet containing 65 mg/kg Mn from Mn_2O_3 nanoparticles preparation per 1 kg of a diet during 12 weeks of feeding.
